# Determination of the Volatile Constituents in *Radix Flemingiae Philippinensis* by GC–MS and a Heuristic Evolving Latent Projection Method

**DOI:** 10.3390/molecules15064055

**Published:** 2010-06-04

**Authors:** Jian-Wei Xie, Wei Hu, Zhao-Liang Zhou, Lan-Fang Huang, Yu-Ling Wang, Jian-Jun Fang, Yun-Biao He

**Affiliations:** 1 Department of Chemistry and Pharmaceutical, Quzhou College, Quzhou 324000, China; 2 Quzhou Center of Calibration and Testing for Quality and Technical Supervision, Quzhou 324000, China; 3 Changde Institute for Drug Control, Changde 415000, China

**Keywords:** chemometrics, heuristic evolving latent projections (HELP), gas chromatography-mass spectrometry (GC–MS), *Radix Flemingiae Philippinensis*, the volatile constituents

## Abstract

With the help of chemometric resolution methods, a technique for qualitative and quantitative determination of the volatile chemical constituents in *Radix Flemingiae Philippinensis* by chromatography-mass spectrometry was developed. After the overlapping chromatographic peaks were resolved into pure chromatograms and spectra using a heuristic evolving latent projections (HELP) method, qualitative analysis was performed by similarity search of the obtained pure mass spectrum of each component in the NIST library and the quantitative results were obtained by calculating the total two-way response volume. A total of 63 components were separated and 55 components were identified, accounting for 90.62% of the total content. The main components were farnesol isomer and β-caryophyllene, accounting for 31.33% and 12.60% of the total content, respectively. The obtained results can provide useful information for further study and development of *Radix Flemingiae Philippinensis*.

## Introduction

Due to their low toxicity, high pharmacological activity and low incidence of complications, people have been paying more and more attention to Traditional Chinese Medicines (TCMs) in recent decades [1]. *Radix Flemingiae Philippinensis*, the root of Flemingiae Philippinensis Merr. et Rolfe, belongs to the leguminous plants class. As a TCM, it has been widely used to cure rheumatism, lumbar muscle strains, myasthenia of limbs, injuries from falls, swelling and sore throat, *etc.* [2]. Although *Radix Flemingiae Philippinensis* contains tens or even hundreds of compounds, only a limited number of compounds might be responsible for any observed pharmaceutical or toxic effects. The volatile compounds are the main pharmacological active components in *Radix Flemingiae Philippinensis*. *Radix Flemingiae Philippinensis*, like other TCMs, is very complex, compared with Western medicines [3], which makes the screening and analysis of bioactive components in it extremely difficult. Generally the analysis of the volatile compounds is performed with gas chromatography (GC) and gas chromatography–mass spectrometry (GC–MS) [4]. GC has seen enormous growth due to its speed, simplicity, relatively low cost, and wide applicability as a separation tool. However, in the case of GC, the identification of components is carried out just by means of comparison of retention times with reference compounds. This means that often some components cannot be identified due to the complexity of the matrix or the lack of an appropriate reference material. In addition, retention times have no uniform criteria because they are affected by many factors. Although GC–MS is a useful and powerful method for the analysis of the volatile compounds in *Radix Flemingiae Philippinensis* [5], only 39 components are qualitatively and quantitatively analyzed by GC–MS. The main reason is that the determination is performed only through the direct similarity searches in MS database attached to the GC–MS instruments. There exist at least two serious problems with the GC–MS method. First, the background can not be accurately corrected. Second as the boiling points of components analyzed are close to each other, there are always overlapping peaks although the chromatographic conditions are optimized. In such a case, it is hard to identify the components by direct similarity search in a MS database alone, which can result in wrong conclusions. Therefore, developing a simple and reliable approach for the determination of the volatile constituents in *Radix Flemingiae Philippinensis* is necessary. To date many associated methods have been developed to provide more information for chemical analysis both in chromatographic separation and in spectral identification, such as evolving factor analysis (EFA) [6,7], window factor analysis (WFA) [8], heuristic evolving latent projections (HELP) [9,10], subwindow factor analysis (SFA) [11,12].These methods make resolution of complex systems possible [13,14,15]. In this paper, the volatile compounds in *Radix Flemingiae Philippinensis* were studied with GC–MS and heuristic evolving latent projections (HELP). The developed method can be used for quality control of *Radix Flemingiae Philippinensis*.

## Results and Discussion

### Qualitative analysis

**Figure 1:**
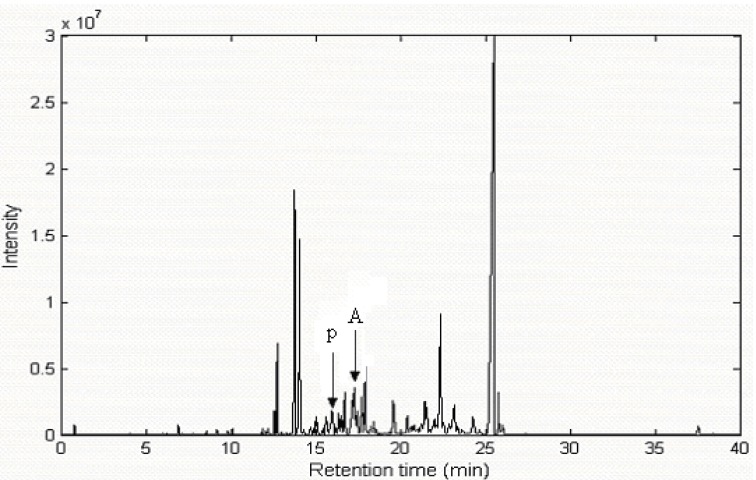
The Total Ion Chromatogram (TIC) of the volatile components in *Radix Flemingiae Philippinensis*.

The real total ion chromatogram (TIC) of the volatile components in *Radix Flemingiae Philippinensis* is shown in Figure 1.

As can be seen from Figure 1, there are a lot of peaks and their relative levels vary greatly. Apparently, it is indeed a very complicated system. Although chromatographic separation was optimized, some of components still overlap with one another and the concentrations of many volatile components are very low. If the compounds were directly searched in the NIST mass spectral database, incorrect identifications may be obtained. First, the similarity indices obtained from direct searching within the NIST mass database were quite low for most of these chromatographic peaks and sometimes the same component was identified at different chromatographic scan points. Second, the components with low content were very difficult to identify directly with the NIST mass database, since two-dimensional data obtained by mass spectral measurement unavoidably contains peaks associated with column background and noise. However, if the overlapped peaks and the components with low content could be resolved into pure spectra and chromatograms with some relative chemometric resolution method, the qualitative analysis of components would be improved to a considerable extent. The HELP method was a commonly used chemometric technique for solving this problem. A brief depiction of the HELP method follows.

**Figure 2:**
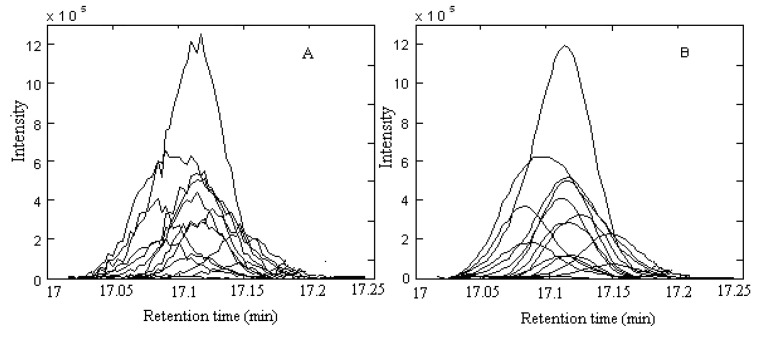
The total ion chromatogram of peak cluster A (A) Data without smoothing; (B) Data after smoothing.

Some data represented by **X**_m×n_ obtained from the GC–MS is a bilinear matrix. According to the Lambert–Beer law, it can be expressed simply as follows:

**X**_m×n_=**CS**^T^+**E=**∑**c**_i_**s**_i_^T^+**E** (i=1,2,…,N) (1)

where **X**_m×n_ denotes an intensity matrix with N components of *m* chromatographic scan points at *n* atom mass units (amu). **C** and **S** are the pure chromatographic matrix and the pure spectral matrix, respectively. The superscript T represents the transpose of matrix **S**. **E** denotes the measurement noise. With local full rank analysis in the HELP method, a two-dimensional data can be resolved into the chromatograms and spectra of the pure chemical components. The TIC of peak cluster A in the 17.0–17.25 min range in Figure 2 is taken as an example to demonstrate the whole procedures of this approach.

**Figure 3:**
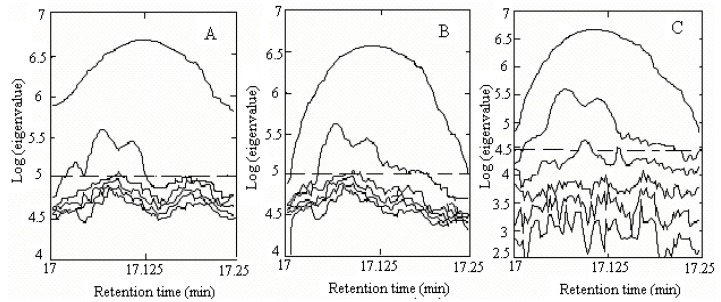
Rank map obtained by FSMWEFA (window size = 6) for cluster A (A) Result of the raw data, (B) result of the data after correcting background and baseline shift, and (C) result of the smoothed data by roughness penalty processing.

First, the background can be estimated by eigenvalue decomposition of the data of the zero component region before and after the chromatographic peak, and then it can be deducted through least squares [13,17]. Then the rankmap was obtained with a fixed size moving wndow evolving factor analysis (FSMWEFA) [18], and was shown in Figure 3.

**Figure 4:**
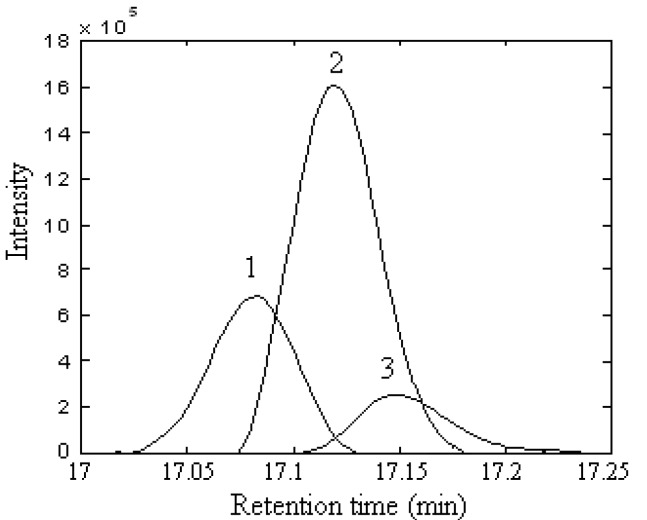
Resolved chromatographic profiles of peak cluster A.(1) calamenene (C_15_H_22_), (2) δ-cadinene (C_15_H_24_),and (3) β-cadinene (C_15_H_24_).

The local rank in the elution sequence can be seen from the rankmap. If the local rank was one, there was one component in the specified region. If the local rank was two, there were two components co-eluting. Figure 3(A) was the rankmap of the raw data with a window size of 6. Apparently it was a kind of typical heteroscedastic noise, the greater the signal, the bigger the noise. Figure 3(B) was the rankmap of the peak cluster A after background and baseline shift corrected. Figure 3(A) and (B) showed that the difference between them mainly came from the 17.0 min to 17.03 min region. In this region, Figure 3(A) seemed to tell us there were two components, but in the same region in Figure 3(B) only one component was shown. In fact, this was caused by the background. We can see the lower signal-to-noise ratio and heteroscedastic noise were still existence in both cases. However, if the data was smoothed by the rough penalty smoothing method [19], the detection ability of the GC–MS data would be improved. Unlike polynomial regression, the roughness penalty approach is a kind of non-parametric regression, which aims to overcome white noise in hyphenated chromatographic data. The chromatograms of peak cluster A after smoothing were shown in Figure 2(B). It was seen that the data has been reasonably smoothed without apparent distortion. Its rankmap was shown in Figure 3(C) and the eigenvalve representing the noise level was on the same level. In Figure 3(C), not only the first and second eigenvalue provide the component information, but the third eigenvalue can also provide the refined structure of the component elution information, the start and end point of each component. Apparently, heteroscedastic noise in the data has been removed and the signal-to-noise ratio was increased with the help of the smoothing treatment. Therefore, the rankmap clearly showed us that there were three components in peak cluster A. With all the information determined, the two-dimensional data matrix can be resolved into the pure chromatographic profiles and mass spectrum of each component with the HELP method. Finally, when each pure spectrum was extracted and the resolved chromatographic profiles of the three components were obtained (Figure 4), then their identification can be done by similarity searches in the NIST mass database.

**Figure 5:**
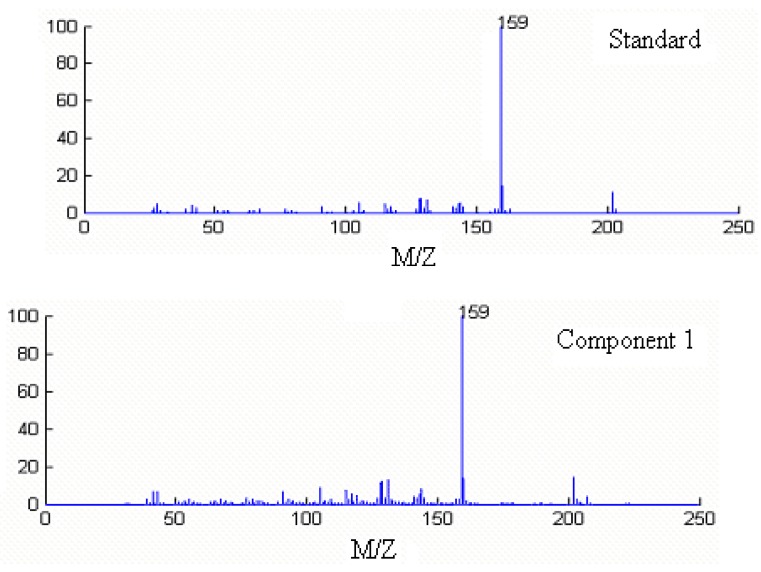
Resolved mass spectrum of component 1 by HELP and standard mass spectrum of calamenene (C_15_H_22_).

**Figure 6:**
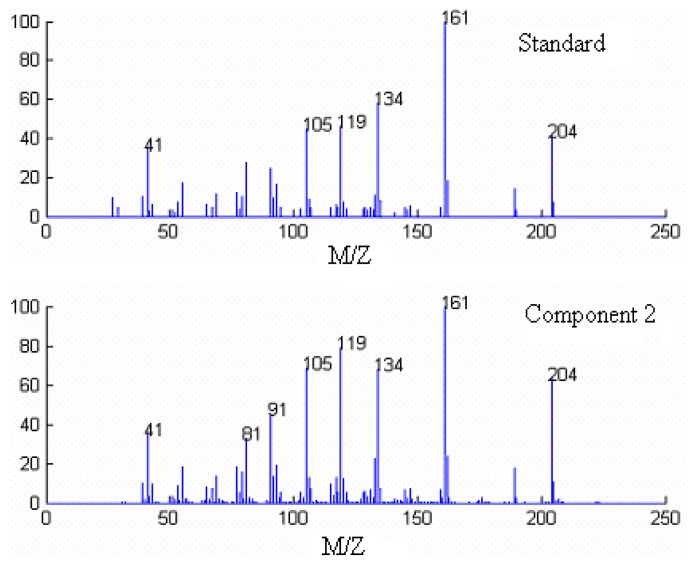
Resolved mass spectrum of component 2 by HELP and standard mass spectrum of δ-cadinene (C_15_H_24_).

**Figure 7:**
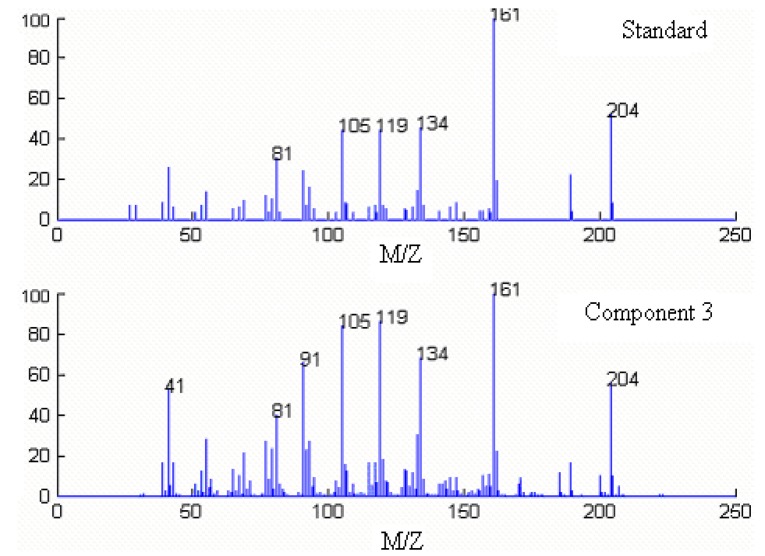
Resolved mass spectrum of component 2 by HELP and standard mass spectrum of β-cadinene (C_15_H_24_).

Component 1, 2 and 3 in this cluster may be calamenene (C_15_H_22_), δ-cadinene (C_15_H_24_) and β-cadinene (C_15_H_24_), with similarity matching indexes of 0.99, 0.97 and 0.93, respectively. The accuracy and reliability of the results were increased greatly. The searched corresponding standard spectra of each component in cluster A were shown in Figure 5, Figure 6 and Figure 7.

**Figure 8:**
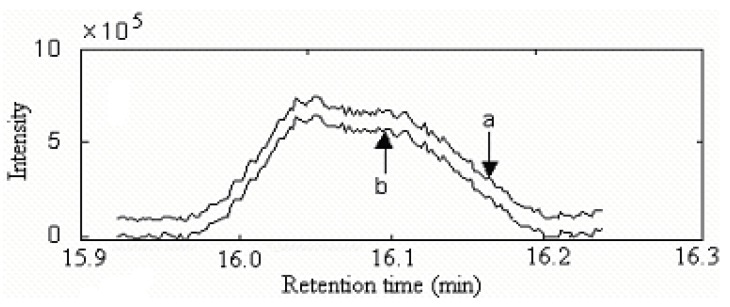
The TIC chromatogram of peak cluster P (a) Curve a was the original chromatogram. (b) Curve b was the chromatogram after background removal.

Other overlapped peaks certainly existed in the total ion chromatogram (TIC) of the volatile components in *Radix Flemingiae Philippinensis*. The TIC of peak cluster p in Figure 1 within 15.9–16.3 min, which was shown in Figure 8, was another example. It seemed to include one component peak. However, in this way, the spectrum changed a lot at different positions. In fact, the similarity was relatively lower. Therefore, it was also necessary to resolve this overlapped peak. Background, baseline shift and heteroscedastic noise in the raw dimensional data was pretreated by the corresponding method [13,17,19].

**Figure 9:**
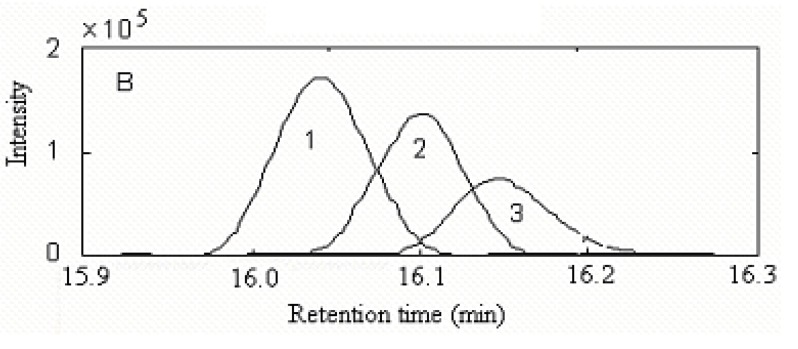
Resolved chromatographic profiles of peak cluster P: (1) 2-cyclopropylidene-1,7,7-trimethylbicylo [2,2,1]heptane (2) 3,3,5,5-tetramethylcyclohexanol and (3) not identified.

**Figure 10:**
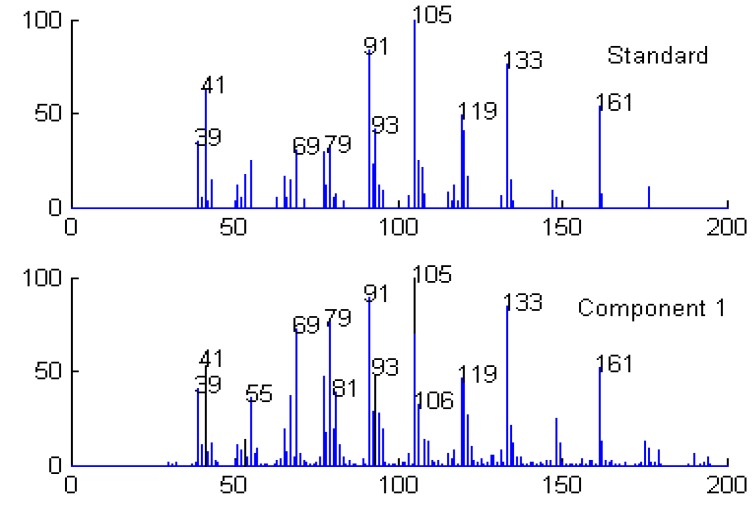
Resolved mass spectrum of component 1 by HELP and standard mass spectrum of 2-cyclopropylidene-1,7,7-trimethylbicylo [2,2,1]heptane (C_13_H_20_).

**Figure 11:**
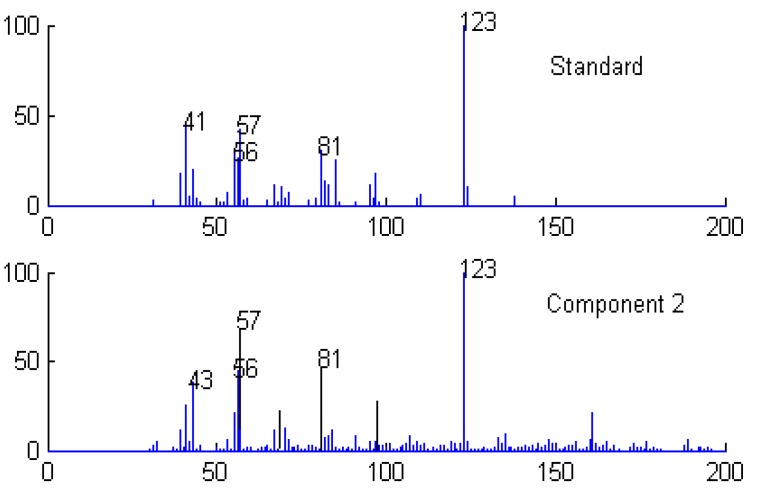
Resolved mass spectrum of component 2 by HELP and standard mass spectrum of 3,3,5,5-tetramethylcyclohexanol(C_10_H_20_O).

According to the rank estimation method [18], there were three components in this peak cluster. With all the information determined, the two-dimensional data matrix can also be resolved into pure chromatographic profiles and mass spectrum of each component with the HELP method. Figure 9 showed the resolved chromatographic profiles. Components 1 and 2 in this cluster may be 2-cyclopropylidene-1,7,7-trimethylbicylo [2,2,1]heptane (C_13_H_20_) and 3,3,5,5-tetra-methylcyclohexanol (C_10_H_20_O), with similarity matching results of 0.97 and 0.95, respectively. The searched corresponding standard spectra of each component in cluster p are shown in Figure 10 and Figure 11. Component 3 were not identified due to the very low match values. Likewise, the spectra of components in other segments can be obtained. The qualitative results were listed in Table 1.

**Table 1:** Identification and quantification of the volatile components in *Radix Flemingiae Philippinensis*.

Series No.	Retention time (min)		Compound name	Molecular formula	Relative content (%)
1	3.653		Hexanal	C_6_H_12_O	0.29
2	6.368		2-Amylfuran	C_9_H_14_O	0.08
3	7.979		Linalool	C_10_H_18_O	0.04
4	8.682		L-camphor	C_10_H_16_O	0.05
5	9.267		*p*-Menth-1-en-4-ol	C_10_H_18_O	0.04
6	9.419		*p*-Menth-1-en-8-ol	C_10_H_18_O	0.13
7	10.549		Nonanoic acid	C_9_H_18_O_2_	0.06
8	11.065		Bornyl acetate	C_12_H_20_O_2_	0.08
9	11.294		2,3,4,5-Tetramethyltricyclo[3.2.1.02,7]oct-3-ene	C_12_H_ 18_	0.11
10	12.750		α-Cubebene	C_15_H_24_	0.13
11	12.929		α-Longipinene	C_15_H_24_	0.11
12	13.035		Di-epi-α-Cedrene	C_15_H_24_	0.04
13	13.330		Ylangene	C_15_H_24_	1.22
14	13.476		Longicyclene	C_15_H_24_	2.49
15	13.675		Copaene	C_15_H_24_	0.06
16	14.315		Longofolene	C_15_H_24_	5.20
17	14.560		β-Caryophyllene	C_15_H_24_	12.60
18	14.744		Germacrene D	C_15_H_24_	0.30
19	15.057		(Z)-β-Farnesene	C_15_H_24_	0.47
20	15.232		2,4a,5,6,7,8,9,9a-Octahydro-3,5,5-trimethyl-9-methylene 1 *H*-benzocycloheptene	C_15_H_24_	0.52
21	15.398		α-Caryophyllene	C_15_H_24_	0.73
22	15.665		Dihydrocurcumene	C_15_H_24_	0.19
23	15.812		α-Curcumene	C_15_H_24_	1.20
24	15.858		Eremophilene	C_15_H_24_	0.56
25	16.037	2-Cyclopropylidene-1,7,7-trimethylbicylo-[2,2,1]- heptane		C_13_H_20_	0.56
26	16.142	3,3,5,5-Tetramethylcyclohexanol		C_10_H_20_O	0.59
27	16.153	Longifolene-(V4)		C_15_H_24_	0.64
28	16.288	α-Guaiene		C_15_H_24_	0.28
29	16.419	Patchoulene		C_15_H_24_	0.33
30	16.551	α-Muurolene		C_15_H_24_	1.69
31	16.721	β-Himachalene		C_15_H_24_	1.59
32	17.092	Calamenene		C_15_H_22_	1.85
33	17.133	δ-Cadinene		C_15_H_24_	2.13
34	17.217	β-Cadinene		C_15_H_24_	0.90
35	17.538	Acoradiene		C_15_H_24_	0.96
36	17.693	3-Bromomethyl- 1,1-dimethyl-1 *H*-indene		C_15_H_22_	3.83
37	18.134	Nerolidol		C_15_H_26_O	1.57
38	18.255	Cadala-1(10),3,8-triene		C_15_H_22_	0.26
39	18.710	Caryophyllenyl alcohol		C_15_H_26_O	0.18
40	19.076	Caryophyllene oxide		C_15_H_24_O	1.37
41	19.772	Cedrol		C_15_H_26_O	1.94
42	19.969	Bulnesol		C_15_H_26_O	0.31
43	20.442	Epiglobulol		C_15_H_26_O	0.36
44	20.611	Cubenol		C_15_H_26_O	2.67
45	20.996	ι-Cadinol		C_15_H_26_O	0.50
46	21.090	δ-Cadinol		C_15_H_26_O	0.75
47	21.196	Torreyol		C_15_H_26_O	0.89
48	21.377	β-Selinenol		C_15_H_26_O	2.33
49	21.510	α-Eudesmol		C_15_H_26_O	0.27
50	22.025	Cadalene		C_15_H_18_	1.34
51	22.185	Hedycaryol		C_15_H_26_O	0.11
52	22.916	Peruviol		C_15_H_26_O	0.71
53	25.654	Farnesol isomer		C_15_H_26_O	31.33
54	26.361	*cis*-Farnesal		C_15_H_24_O	0.66
55	33.868	Hexadecanoic acid		C_16_H_32_O_2_	1.12

### Quantitative analysis

After the pure chromatographic profile and mass spectrum of each component were resolved, the total two-way response of each component can be obtained from the outer product of the concentration vector and the spectrum vector for each component, namely **C*_i_*** **S*_i_****^T^*. Similar to the general chromatographic quantitative method with peak area or height, the concentration of each component is proportional to the overall volume of its two-way response (**C*_i_*** **S*_i_****^T^*). The advantage of this quantitative method over general peak-area integration is that all mass spectral absorbing points are taken into consideration. The final relative quantitative results were also listed in Table 1. A total of 55 components were identified in *Radix Flemingiae Philippinensis,* which represented 90.62% of the volatile components.

## Experimental

### Apparatus

GC–MS was performed with Shimadzu GC-2010 gas chromatography instrument coupled with a Shimadzu 2010 mass spectrometer. Compounds were separated on a 30 m × 0.25 mm i.d. capillary column coated with 0.25μm OV-1 film.

### Materials

*Radix Flemingiae Philippinensis* was purchased from a Zhejiang pharmaceutical store market and was identified by a researcher from Institute of Materia Medica, Zhejiang Academy of Traditional Chinese Medicine and Materia Medica.

### Extraction of the essential oil

The samples were dried at constant temperature 40 °C for 2 h, and extracted by water distillation for 8h, using a set of standard apparatus, according to the procedure described in the Chinese Pharmacopoeia [16]. The essential oils obtained were stored in the refrigerator at 4 °C prior to analysis. The yield of the essential oil samples of *Radix Flemingiae Philippinensis* was 0.35% (*v/w*).

### Gas chromatography–mass spectrometry

Analytical conditions were as follows: the oven was held at 50 °C for 1 min during injection, then temperature programmed at 10 °C·min^-1^ to 150 °C and then ramped at 2 °C·min^-1^ to a final temperature of 200 °C and held for 5 min. Inlet temperature was kept at 260 °C all the time. A 1.0 µL volume of essential oil was injected into the GC. Helium carrier gas at a constant flow-rate of 1.0 mL·min^-1^ and a 10:1 split ratio were used simultaneously. Mass spectrometer was operated in full scan and electron impact (EI^+^) modes with an electron energy of 70eV. Interface temperature was 250 °C and MS source temperature was 200°C. In the range of m/z 30 to 350, mass spectra were recorded with 0.2 s·scan^-1^ velocity.

### Data analysis

Data analysis was performed on a Pentium based IBM compatible personal computer. All programs of the chemometrics resolution methods were coded in MATLAB 6.5 for Windows. The library searches and spectral matching of the resolved pure components were conducted on the National Institute of Standards and Technology (NIST) MS database, containing about 107,000 compounds.

## Conclusions

In this work, the volatile constituents in *Radix Flemingiae Philippinensis* have been determined with GC-MS and a combined chemometrics resolution method for the first time. Fifty five of 63 separated constituents in the essential oil of *Radix Flemingiae Philippinensis* were identified and quantified, accounting for 90.62% of the total content. The developed method can be used for the identification, differentiation and quality evaluation of *Radix Flemingiae Philippinensis*. The obtained results showed that the combination of GC-MS and a relative chemometrics resolution method are a useful and powerful technique for the analysis of bioactive components in TCMs.
